# Measurement of the Equality of the Drug Welfare Induction Level of Chinese Patients With Chronic Diseases in Gansu, Sichuan, Hebei, and Zhejiang Based on the Bivariate Theil-T Index Method

**DOI:** 10.3389/fpubh.2020.581533

**Published:** 2020-10-28

**Authors:** Shaoliang Tang, Ruxia Zhang, Yinghang Si, Yan Cheng, Ying Gong

**Affiliations:** School of Health Economics and Management, Nanjing University of Chinese Medicine, Nanjing, China

**Keywords:** patients with chronic diseases, drug welfare induction, bivariate Theil-T index, measurement of equalization, health equality

## Abstract

**Objectives:** This study aimed to measure the induction level of drug welfare in Chinese patients with chronic diseases using a bivariate Theil index.

**Design:** The bivariate Theil-T index was used to hierarchically decompose the relevant survey data, and the contribution rate of the intragroup gap and the intergroup gap to the total gap was investigated to better understand the current drug welfare induction level of Chinese patients with chronic diseases.

**Setting:** The study was based in Gansu, Sichuan, Hebei, and Zhejiang provinces in China.

**Participants:** Survey data was from patients with chronic diseases in 20 hospitals in four provinces.

**Primary and secondary outcome measures:** Data was collected through a questionnaire designed by the research team after expert consultation. Using the variables represented by the index system to decompose the Theil index from the two dimensions of the region and urban and rural areas. SPSS 22.0 was used for reliability and validity analysis and Theil index calculation.

**Results:** The overall level of drug welfare induction in Chinese patients with chronic diseases had a high degree of equalization. The overall Theil index was 0.0003, but there were still some differences among groups.

**Conclusions:** To improve the drug welfare equalization induction level of patients with chronic diseases in China, the government should start from western rural areas, and policy should target the provinces that were in a disadvantaged position within the region to promote the equalization of drug welfare induction level for patients with chronic diseases in China.

## Introduction

With the change of the human disease spectrum, chronic diseases currently account for the majority of global morbidity, and mortality (1, 2). It is expected that by 2020, chronic non-communicable diseases (chronic diseases) will become the leading cause of death and disability in humans (3, 4) and the most crucial disease burden in China. Chronic diseases have brought many challenges and burdens to patients and the health system. Whether the drug needed for treatment is obtained and affordable is the most significant way to effectively control the incidence and mortality of chronic diseases.

Chronic diseases, also known as non-communicable diseases (NCDS), are ongoing and often incurable diseases or conditions that require ongoing medical care and affect a person's daily life (5, 6). It reported that 80–92% of older adults have at least one chronic illness and 50–77% have two or more (7). Cardiovascular disease, arthritis, and diabetes are common chronic diseases. Some studies indicate that heart disease and cancer together account for nearly 46% of all deaths^.^(8). Older people with arthritis found that they have trouble with their normal activities (9, 10). Diabetes is the leading cause of kidney failure, lower-limb amputation not due to injury, and new cases of blindness among adults (11). Moreover, it has also been reported that ~63% of all deaths in the world are attributed to NCDs, and this causes great socioeconomic harm to all countries, particularly developing countries (12).

The disease characteristics of chronic diseases determine that patients with chronic diseases need to take drugs for a long time, and the cost of medicines accounts for the vast majority of medical expenses. Due to the fragility of their social status, chronically ill patients are often at the most disadvantaged position for access to basic health services, especially medicines, which will directly affect their health and drug welfare effects. The expenditure of chronic disease drugs brings many challenges and burdens to the patients themselves and the health system (13). Whether the drugs needed for treatment can be obtained and can afford them is the most important to effectively control the incidence and mortality of chronic diseases. In addition, the equalization of basic public goods is an important goal for the future development of Chinese society. As a special commodity, medicines belong to the category of quasi-public products, and ensuring the fairness of medicines is an important basis for achieving the health of the whole people.

The Chinese government is aware of the seriousness of this problem. Since the new medical reform in 2009, the state has successively introduced and implemented relevant drug policies, such as the basic drug system, the zero-rate policy for essential drugs, and the centralized procurement of drugs in public hospitals. It is of vital importance to improve the level of drug welfare in Chinese patients. One of the landmark initiatives is the issuance of a national essential medicines system with the specific goal of improving the supply of medicines and ensuring equitable access to essential medicines. Since the implementation, the overall burden of medicine for patients with chronic diseases has improved (14), but it still faces a severe situation. The survey showed that patients with type 2 diabetes (T2DM) who used oral antidiabetic drugs (OADs) in China had a heavy financial burden, with direct treatment costs and opportunity costs accounting for 56% of the patient's disposable income (15).

The main contributions of this research include three aspects: First, due to the widespread urban-rural differences and regional differences in China, and the different socio-economic systems in different regions, we have carried out the level of drug welfare sensing for chronically ill patients through the hierarchical decomposition of the bivariate Theil index. Dimensional research. Secondly, we make full use of the survey data to reflect the actual situation more intuitively, and try to study the equalization of the drug welfare level from the perspective of Chinese chronic disease patients. Third, we seek to provide empirical support to policy makers to develop more effective drug policies and establish more effective public health management systems.

The rest of this article is organized as follows. The second part describes the samples and research methods. The third part analyzes the decomposition results of the bivariate Theil index. The fourth part discusses the empirical results. The fifth section summarizes the main conclusions.

## Backgrounds in Drug Welfare

Domestic and foreign research on “drug welfare” generally refers to “Pharmacy Benefit Management” (PBM), which is a management coordination organization between insurance institutions, pharmaceutical companies, hospitals, and pharmacy. PBMs have the potential to secure lower drug prices and to improve rational prescribing (16). PBMs decides on the use of medicines in formulas and negotiates with pharmaceutical manufacturers and pharmacies on behalf of insurance companies. Through these activities, PBMs provides value by curbing drug spending (17).

Grabowski and Mullins analyzed the cost-effectiveness of drug welfare management in the United States, and proposed that PBM as a drug welfare management agency, through the control of hospital medical expenses and doctor behavior, control of medical insurance costs and the main profit of the pharmaceutical supply chain, the most important purpose was to improve patient utility (18). Tara L. Jenkins conducted a retrospective administrative analysis using the Oklahoma Health Care Pharmacy and Medical Claims Database, and the results showed although total health care expenditures increased after a monthly pharmacy benefit in a Medicaid population was expanded, a subpopulation of recipients identified as high pharmacy users before the expansion did not have a statistically significant increase in medical expenditures, and their pharmacy welfare status had not been significantly improved (19). Sean et al. explored the reasons why the private sector in Canada was unable to implement prescription drug cost control measures through semi-structured telephone interviews with relevant experts, and suggested that employees and employers needed to be educated to have more collaboration and data sharing between each other. It also required external government intervention to help transform the established norms of private drug program design with a view to improving employee private drug benefits (20). Lohrberg et al. analyzed the definition and role of QoL in German drug welfare assessment, and the use of QoL as a drug welfare standard emphasized the importance of defining QoL definitions and methodological regulations (21).

By combing the literature, it can be found that the research on drug welfare in the academic community mostly starts from the single dimension of the fairness of health resources, and lacks multi-dimensional exploration. We reviewed the relevant literature on the evaluation of drug welfare effects. Based on relevant theories and methods, combined with the characteristics of prevention and treatment of chronic diseases, and the uniqueness of patients with chronic diseases, from the perspective of multi-dimensional research, we studied the drug welfare induction level of chronic disease patients from four aspects: drug accessibility sensing level, drug price sensing level, drug fairness sensing level, and drug health sensing level.

## Methods

### Measuring Instruments

The National Natural Science Foundation project hosted by the author, “Optimization of Health and Precision Poverty Alleviation Policy Based on the Improvement of the Drug Welfare Effect of Poverty Chronic Patients,” has made a detailed study on the construction of the evaluation index system for the drug welfare level of patients with chronic diseases in China [Min (22) “Study on the Evaluation and Promotion Strategy of the Drug Welfare Effect of Patients with Chronic Diseases in 10 Provinces Based on the Topsis Method;” Yini (2018) (23) “Study on the Drug Welfare Effect of Patients with Chronic Diseases Based on the Two-Step Clustering Method”]. So according to the four aspects of patients' drug welfare induction level mentioned before, this study established an evaluation index system for drug welfare effects of chronic diseases patients (Table 1). The weight of each index was obtained by Delphi expert consultation method. The evaluation index system is shown in Table 1 and will not be discussed in this paper.

**Table 1:** Weighting evaluation system for equalization of drug welfare induction in patients with chronic diseases.

**Target layer**	**Index level one**	**Index level two**	**Weights**
X: Drug welfare effect induction level equalization	A1: Drug accessibility effect induction level equalization	I1: The number of medical service institutions that can be reached within 15 min	0.1064
		I2: The needed drug can be purchased in public medical heath institutions	0.1127
		I3: The needed drug can be purchased in retail pharmacies	0.1032
		I4: The needed drug can be purchased in online pharmacies	0.0694
		I5: The cheap needed drug is not accessible	0.3105
		I6: The expensive needed drug is not affordable	0.2978
	A2: Drug price effect induction level equalization	I7: The percentage of drug expenditure out of household disposable income	0.2664
		I8: Drug expenditure spent in public health institutions	0.2518
		I9: Drug expenditure spent in retail pharmacies	0.1275
		I10: Drug expenditure spent in online pharmacies	0.0203
		I11: The percentage of outpatient drug expenses	0.1956
		I12: The percentage of hospitalization drug expenses	0.1384
	A3: Drug fair effect induction level equalization	I13: Medical insurance reimbursement level of medical expenses	0.2732
		I14: Second reimbursement level of drug expenses	0.2537
		I15: Level of self-paying drug affordability	0.2177
		I16: Resident satisfaction of prescriptions	0.0725
	A4: Drug health effect induction level equalization	I17: EQ-5D-5L rating scale	0.6896
		I18: BMI	0.3104

### Design of Questionnaire

According to Table 1, the questionnaire on the drug welfare effect of patients with chronic diseases was compiled, which consisting of five parts: (1) Personal situation questionnaire (2) Drug accessibility questionnaire (3) Drug price effect questionnaire (4) Drug Fair Effect Questionnaire (5) European five-dimensional health scale. Except for personal situation, the other four parts were based on the chronic disease patients in Table 1.

- Part 1 included the basic sociodemographic information of hukou nature, gender, ethnicity, age, marital status, education level, occupation, average monthly income (yuan), type of medical insurance, employment status, current main source of income, health file, health checkup, types of chronic disease.
- Part 2 has six questions for knowing about the situation of drug accessibility induction level equalization.
- Part 3 has six questions for knowing about the situation of drug price effect induction level equalization.
- Part 4 has four questions for knowing about the situation of drug fair effect induction level equalization.
- Part 5 has five questions for knowing about the situation of drug health effect induction level equalization.

There are positive indicators and inverse indicators in the questionnaire. For the positive indicators, the larger the score is, the better the indicators. For the inverse indicators, the smaller the score is, the better the indicators. For the inverse indicators, the method of taking the reciprocal of the original indicator is adjusted. There are two reverse indicators in this article, namely, “I5: The cheap drugs needed are not accessible and I6: The expensive drugs needed are not affordable.” This article has made relevant adjustments before using the data, and the rest of the indicators are all positive indicators. The questionnaire is detailed in Appendix 1.

### Participants and Sampling

Our research was conducted in both eastern and western region in China, provinces with higher and lower per-capita gross domestic product were sampled. In 2018, Zhejiang province was ranked the fifth in terms of per capita GDP among the 31 provinces or municipalities in the mainland in China, with a GDP per capita of 99,000 yuan. Hebei province was the 20 th(48,000 yuan). Among the eastern regions, we selected Zhejiang province and Hebei province as the sample area with relatively high and low economic level among the eastern regions, which could better represent the areas in eastern China. At the same time, in the western region, Sichuan province, and Gansu province were ranked the 18 th (49,000 yuan) and 28 th (31,000 yuan) on per capita GDP, which could better represent the areas in western China.

Thus, four provinces were selected, two from each of these two regions. In total, 20 hospitals were sampled in our study. Each province was sampled according to their geographical distribution, and five hospitals were then randomly selected from each province. In each hospital, trained data collectors went to check patients' messages. After each interview, which lasted ~15 min, the participant received a small gift of thanks. Approximately 50 patients per hospital were selected for this study. Through questionnaire survey, relevant data of patients with chronic diseases were obtained, and informed verbal consent was obtained from the respondents; according to the scope of the items covered by the questionnaire, a face-to-face questionnaire survey was conducted with individuals who were in the hospitals. Finally, Members of 1,000 patients were interviewed and the response rate was 99.2%.

A total of 992 patients reported their messages about drug welfare induction level, of which 29 were excluded because they were not having chronic diseases. The total number of residents included in the analysis was 963. We used the simple random sampling formula to calculate the sample size of chronic disease patients in four provinces, because we only had limited literature and more parameters were needed in the stratified sample size calculation formula (24). After we got the total sample needed, we allocated the sample size in selected hospitals using probability proportionate to size sampling (PPS) (25).

The simple random size calculation formula (26):

$$N=\frac{Z_{\alpha }^{2}*\pi (1-\pi )}{E^{2}}$$

Let E = 0.05, Z = 1.96, α = 0.05,π(1-π)= 0.5. Taking incomplete questionnaires into account, we should survey about 384 chronic disease patients. Obviously, the sample size of this study meets the requirements.

This study used SPSS 22.0 software to summarize the results of the 963 questionnaires collected and performed reliability and validity analysis. The results of the study showed that the Cronbach's α value of the 18 evaluation indicators involving the equalization of drug welfare induction in patients with chronic diseases was 0.733, and the Cronbach's α value based on the standardized project was 0.741, both of which were >0.7. Therefore, the questionnaire indicators may be considered to have high validity. The KMO value was 0.791, and the *p* < 0.001, further indicating that the questionnaire was valid.

### Measurements

**Figure 1:**
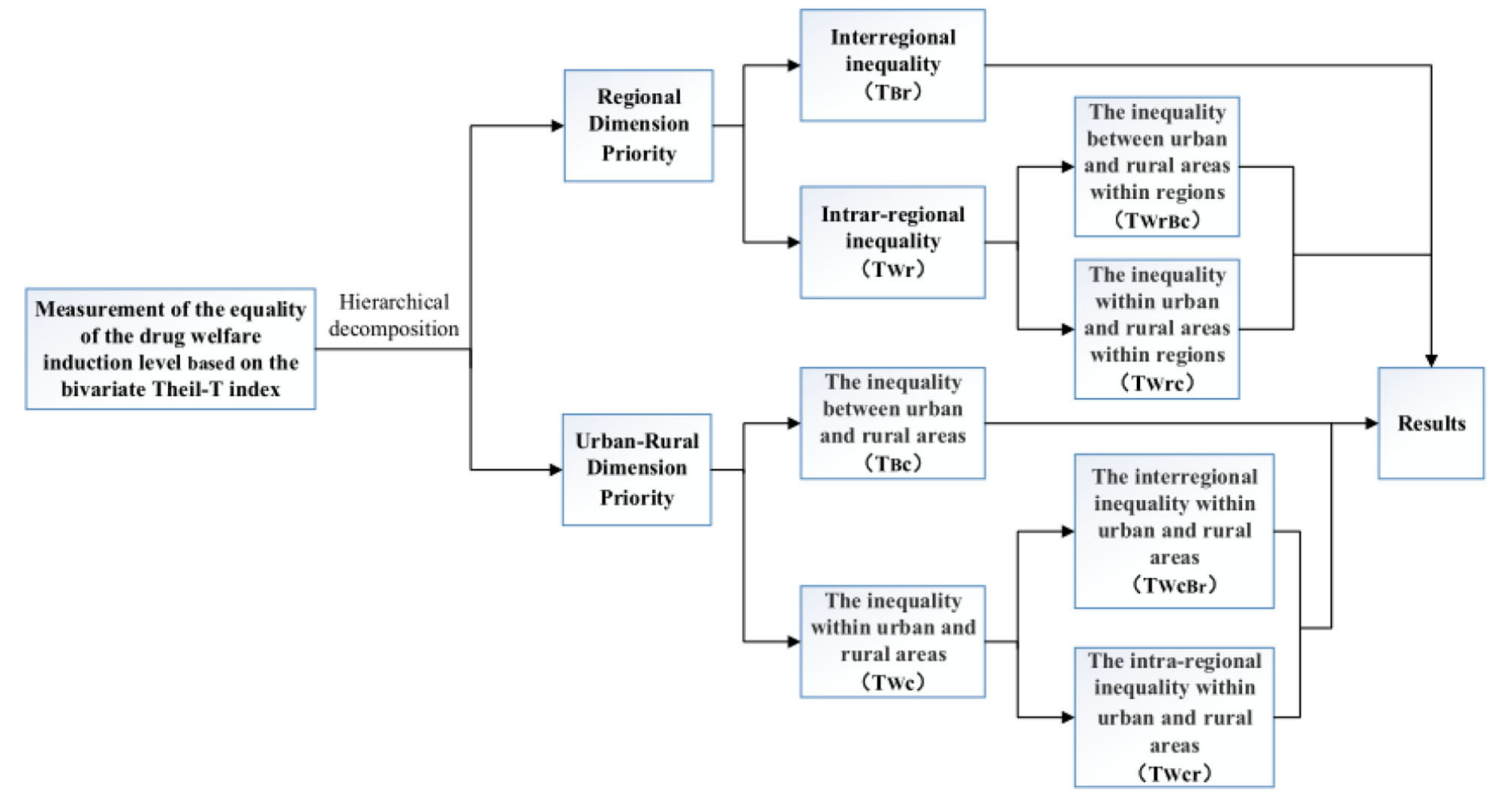
Decomposition of bivariate Theil-T index of drug welfare induction level in patients with chronic diseases.

We first use the weighted summation method to evaluate the level of equalization. Then the bivariate Theil-T index is used to decompose it hierarchically. Through layer decomposition, we can examine the overall difference structure from different perspectives. The contribution rate of the intra-group gap and the inter-group gap to the total gap can better understand the current equalization of the drug welfare level of chronic disease patients in China. See Figure 1 for details.

### Theil Index: Definition and Decomposition

The statistical methods commonly used to measure the level of equalization of pharmaceutical welfare (public service) include the coefficient of variation method, the Gini coefficient method, and the Theil index method. All of the above methods can provide relatively scientific reference results to a certain extent, but it is difficult to accurately reflect the difference in the level of urban-rural equalization or the difference between urban and rural areas. Compared with other equalization measurement tools, the Theil index has obvious technical advantages: First, compared with the Gini coefficient equalization measurement tool, the Theil index equalization measurement tool is more laterally reorganized in the analysis process. The problem of decomposability emphasizes the degree of equalization of the population according to different group criteria. Second, compared with the equalization measurement tool of the coefficient of variation, the measurement index of the Theil index equalization can achieve unevenness in the measurement process. Steady reduction, more focused on the analysis of conduction sensitivity problems in the process of unequal (27).

The Theil T index (28) was used to quantify inequality at a district level. This index has been widely used to measure inequality in different health and social outcomes. For example, it has been used to measure income inequality in Latin America (29) or inequality in access to improved water in different world regions (30). Theil-T is a population weighted index that is sensitive to health differences further from the average rate (31).

In China, urban-rural differences and regional differences are large, and the use of the bivariate Theil index has innovative practical significance. At the same time, combining the two variables can examine the combined effect of the two on the level of equalization. Based on comprehensive considerations, this paper uses the bivariate Theil- T index in the Theil index equalization measurement tool to equalize the drug welfare induction level of chronic disease patients in China.

Due to its decomposability, the Theil index can be decomposed by intraregional differences and interregional differences, thereby measuring the contribution of intraregional differences and interregional differences to the total differences (32–36). For this study, the Theil-T index refers to the degree of unequal distribution of drug welfare in patients with chronic diseases in different regions or in urban or rural areas relative to the population, that is, the perceived degree of unequal drug welfare per capita.

### Bivariate Theil-T Index With Regional Dimension Priority

**Figure 2:**
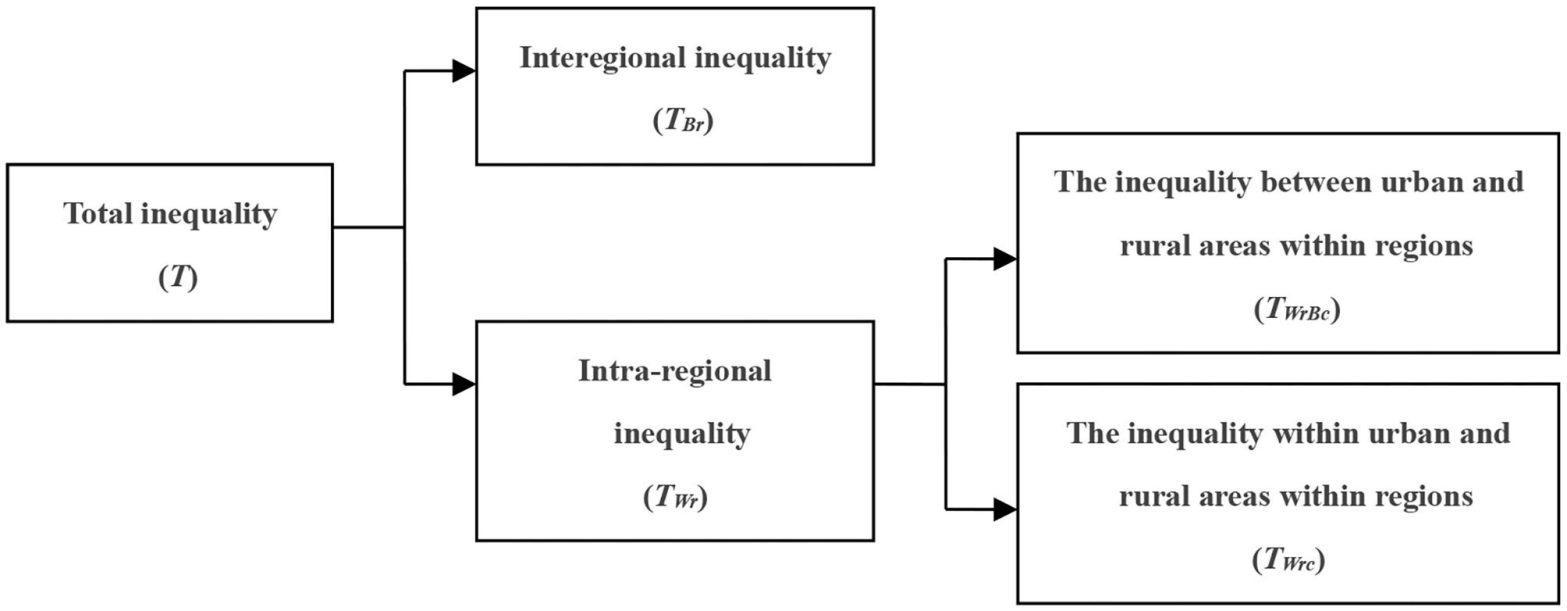
Decomposition diagram along the regional dimension.

The bivariate Theil-T index adopts with priority to the regional dimension; that is, all the unequal indicators expressed by the Theil index are first decomposed according to the regional dimension. The first level is decomposed into interregional inequality (between western region and eastern region) and intra-regional inequality (within western region or within eastern region). At the second level, the intra-regional inequality is decomposed into the inequality between urban and rural areas within regions, and the inequality within urban and rural areas within regions (37, 38), which is shown in Figure 2.

This paper uses four provinces of China (Gansu Province, Sichuan Province, Hebei Province, Zhejiang Province) as the basic research unit. The four provinces are divided into two groups according to geographical regions: the western region and the eastern region. The provinces included in each region are shown in Table 2.

**Table 2:** The two major regional divisions of China and representative provinces included.

**Region**	**Representative provinces**
Western region	Gansu, Sichuan
Eastern region	Hebei, Zhejiang

Accordingly, the bivariate Theil-T index with hierarchical decomposition, giving priority to the regional dimension, is expressed as follows (39):

$$\begin{array}{l} T=T_{B_{r}}+T_{W_{r}}=T_{B_{r}}+T_{W_{r}B_{c}}+T_{W_{rc}}\\ =\sum_{r}(\frac{Y_{r}}{Y}\log\frac{Y_{r}/Y}{N_{r}/N})+\sum_{r}(\frac{Y_{r}}{Y}(\sum_{c}(\frac{Y_{rc}}{Y_{r}}\log\frac{Y_{rc}/Y_{r}}{N_{rc}/N_{r}})))\;(\text{Formula1})\\ +\sum_{r}\sum_{c}(\frac{Y_{rc}}{Y}(\sum_{i}(\frac{Y_{rci}}{Y_{rc}}\log\frac{Y_{rci}/Y_{rc}}{N_{rci}/N_{rc}}))) \end{array}$$

where *T* is the total Theil index, which measures the overall degree of inequality; *T*_*Br*_ and *T*_*Wr*_, respectively, indicate the degree of inequality of drug welfare induction in patients with chronic diseases among and within regions; *T*_*WrBc*_ and *T*_*Wrc*_, respectively, indicate the degree of inequality of drug welfare induction in patients with chronic diseases between urban and rural areas and between provinces and towns; *r, c*, and *i*, respectively, represent the regional grouping (east and west), urban and rural, and provinces in the *r* region (Gansu Province, Sichuan Province, Hebei Province, and Zhejiang Province), total population of the sample area of the *N*_*rci*_ representative group, and total level of drug welfare induction of the patients with chronic diseases in the sample area of the *Y*_*rci*_ representative group, where:

$$\begin{array}{l} N_{r}=\sum_{c}N_{rc},N_{rc}=\sum_{i}N_{rci},Y_{r}=\sum_{c}Y_{rc},Y_{rc}=\sum_{i}Y_{rci},Y\\ \;=\sum_{r}\sum_{c}\sum_{i}Y_{rci},N=\sum_{r}\sum_{c}\sum_{i}N_{rci}. \end{array}$$

The values of the five indicators *T, T*_*Br*_, *T*_*Bc*_, *T*_*Wrc*_, and *T*_*Irc*_ c are between 0 and 1. The larger the value is, the lower the degree of equalization. The smaller the value is, the higher the degree of equalization.

### Bivariate Theil-T Index of Urban-Rural Dimension Priority

**Figure 3:**
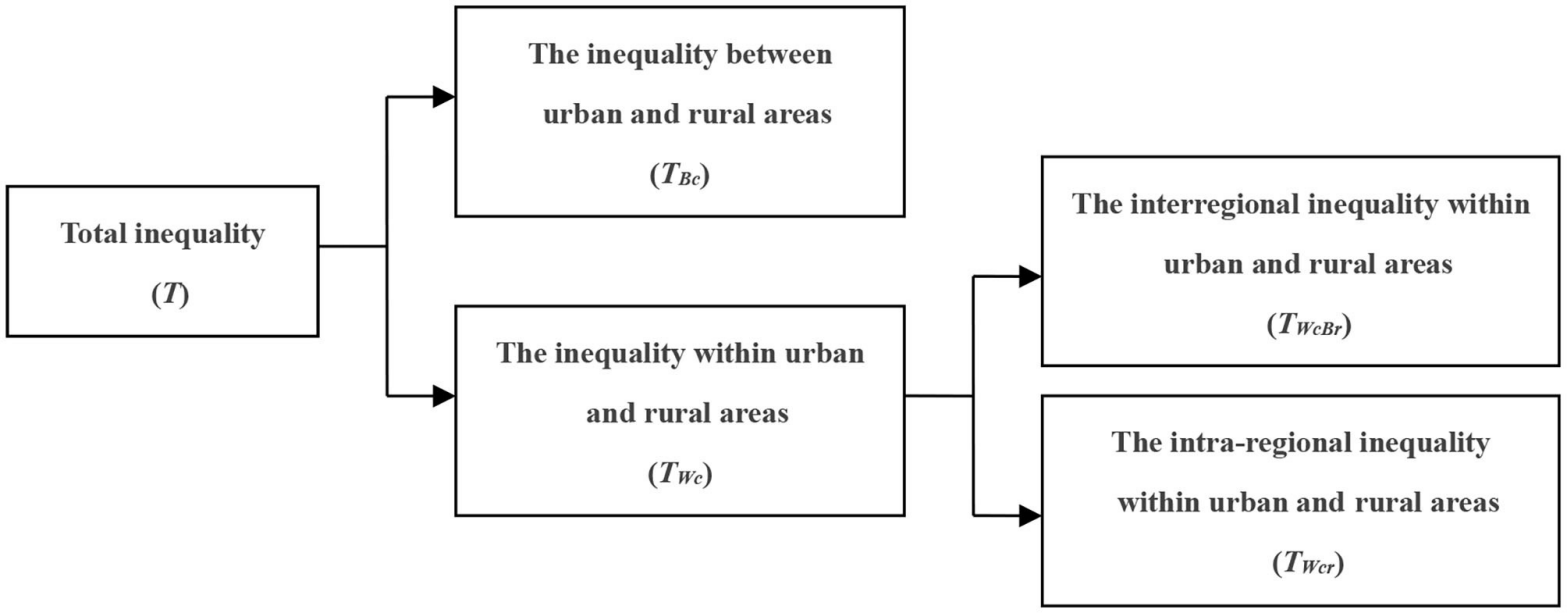
Decomposition diagram along the urban and rural dimension.

This decomposition method uses the Theil index method to measure the equalization of drug welfare in patients with chronic diseases. At the first level, the decomposition concerns the inequality between urban and rural areas and the inequality within urban and rural areas. At the second level, the inequality within urban and rural areas is decomposed into the interregional inequality within urban and rural areas and the intra-regional inequality within urban and rural areas, as shown in Figure 3.

Thus, the bivariate Theil-T exponential decomposition formula that prioritizes the urban-rural dimensions is:

$$\begin{array}{l} T=T_{B_{c}}+T_{W_{c}}=T_{B_{c}}+T_{W_{c}B_{r}}+T_{W_{cr}}\\ =\sum_{c}(\frac{Y_{c}}{Y}\log\frac{Y_{c}/Y}{N_{c}/N})+\sum_{c}(\frac{Y_{c}}{Y}(\sum_{r}(\frac{Y_{cr}}{Y_{c}}\log\frac{Y_{cr}/Y_{c}}{N_{cr}/N_{c}})))\;(\text{Formula}\;\text{2})\\ +\sum_{c}\sum_{r}(\frac{Y_{cr}}{Y}(\sum_{i}(\frac{Y_{cri}}{Y_{cr}}\log\frac{Y_{cri}/Y_{cr}}{N_{cri}/N_{cr}}))) \end{array}$$

where *T*_*Bc*_ and *T*_*Wc*_ indicate the degree of inequality of drug welfare in patients with chronic diseases between urban and rural areas, respectively. *T*_*WcBr*_ and *T*_*Wcr*_ indicate the degree of inequality of drug welfare of patients with chronic diseases in urban and rural areas in different regions, respectively. The other variables have the same meaning as above. Thus,

$$\begin{array}{l} N_{c}=\sum_{r}N_{cr},N_{cr}=\sum_{i}N_{cri},Y_{c}=\sum_{r}Y_{cr},Y_{cr}=\sum_{i}Y_{cri},\\ Y=\sum_{c}\sum_{r}\sum_{i}Y_{cri},N=\sum_{c}\sum_{r}\sum_{i}N_{cri} \end{array}$$

## Results

### Bivariate Theil-T Index Hierarchical Decomposition Calculation of Drug Benefits in Patients With Chronic Diseases—The Regional Dimension

By substituting the research data into formula 1, the hierarchical decomposition results of the bivariate Theil-T index with priority to the regional dimension can be obtained (see Table 3).

**Table 3:** Theil index decomposition results of perceived drug welfare level in patients with chronic diseases with regional dimension priority.

	**I1**	**I2**	**I3**	**I4**	**I5**	**I6**	**I7**	**I8**	**I9**	**I10**	**I11**	**I12**	**I13**	**I14**	**I15**	**I16**	**I17**	**I18**	**Total**
*T*	0.0050	0.0005	0.0004	0.0033	0.0033	0.0046	0.0016	0.0009	0.0004	0.0013	0.0010	0.0007	0.0016	0.0054	0.0025	0.0016	0.0002	0.0001	0.0003
*T_*Br*_*	0.0000	0.0000	0.0002	0.0010	0.0007	0.0015	0.0000	0.0000	0.0000	0.0008	0.0001	0.0000	0.0000	0.0005	0.0000	0.0000	0.0000	0.0000	0.0001
Weighted logarithm (western)	−0.0031	−0.0018	0.0065	−0.0143	−0.0119	−0.0174	−0.0027	−0.0029	−0.0027	−0.0126	−0.0037	−0.0003	0.0023	−0.0099	0.0025	0.0001	0.0000	0.0021	−0.0034
Weighted logarithm (eastern)	0.0031	0.0018	−0.0063	0.0153	0.0126	0.0189	0.0027	0.0029	0.0027	0.0133	0.0038	0.0003	−0.0023	0.0104	−0.0025	−0.0001	0.0000	−0.0020	0.0035
*T_*Wr*_*	0.0049	0.0005	0.0002	0.0023	0.0026	0.0031	0.0016	0.0008	0.0004	0.0005	0.0009	0.0007	0.0016	0.0049	0.0025	0.0016	0.0002	0.0001	0.0002
*T_*WrBc*_*	0.0023	0.0002	0.0001	0.0008	0.0000	0.0004	0.0003	0.0004	0.0001	0.0001	0.0003	0.0004	0.0007	0.0007	0.0008	0.0004	0.0001	0.0000	0.0001
Urban and rural areas in the western region	0.0007	0.0002	0.0000	0.0008	0.0000	0.0002	0.0000	0.0004	0.0000	0.0001	0.0003	0.0003	0.0003	0.0003	0.0000	0.0004	0.0000	0.0000	0.0000
Urban and rural areas in the eastern region	0.0016	0.0000	0.0001	0.0000	0.0000	0.0002	0.0003	0.0000	0.0000	0.0000	0.0001	0.0001	0.0004	0.0004	0.0008	0.0000	0.0001	0.0000	0.0001
*T_*Wrc*_*	0.0026	0.0003	0.0001	0.0015	0.0026	0.0027	0.0013	0.0004	0.0003	0.0004	0.0006	0.0003	0.0009	0.0042	0.0016	0.0013	0.0000	0.0000	0.0002
Rural interior	0.0023	0.0000	0.0001	0.0012	0.0011	0.0010	0.0013	0.0002	0.0001	0.0002	0.0002	0.0003	0.0007	0.0020	0.0011	0.0005	0.0000	0.0000	0.0000
Rural interior of western region	0.0000	0.0000	0.0001	0.0009	0.0001	0.0005	0.0000	0.0000	0.0001	0.0000	0.0001	0.0001	0.0004	0.0000	0.0001	0.0000	0.0000	0.0000	0.0000
Rural interior of eastern region	0.0023	0.0000	0.0000	0.0003	0.0010	0.0005	0.0012	0.0001	0.0000	0.0002	0.0001	0.0002	0.0003	0.0019	0.0010	0.0005	0.0000	0.0000	0.0000
Urban interior	0.0003	0.0002	0.0000	0.0003	0.0015	0.0017	0.0001	0.0002	0.0002	0.0002	0.0004	0.0000	0.0002	0.0022	0.0006	0.0008	0.0000	0.0000	0.0001
Urban interior of western region	0.0001	0.0001	0.0000	0.0000	0.0005	0.0011	0.0000	0.0002	0.0000	0.0000	0.0004	0.0000	0.0001	0.0001	0.0006	0.0003	0.0000	0.0000	0.0001
Urban interior of eastern region	0.0002	0.0001	0.0000	0.0003	0.0010	0.0007	0.0000	0.0000	0.0002	0.0001	0.0000	0.0000	0.0001	0.0021	0.0000	0.0005	0.0000	0.0000	0.0000
*The proportion of T_*Br*_*(%)	0.90	3.32	49.41	30.12	20.92	32.83	2.13	4.63	7.79	59.46	6.41	0.06	1.55	8.86	1.16	0.00	0.00	25.91	18.53
*The proportion of T_*Wr*_*(%)	99.10	96.68	50.59	69.88	79.08	67.17	97.87	95.37	92.21	40.54	93.59	99.94	98.45	91.14	98.84	100.00	100.00	74.09	81.47
*The proportion of T_*WrBc*_*(%)	46.32	42.56	20.82	23.57	0.23	8.56	16.93	50.32	19.23	9.62	31.67	55.61	45.06	12.85	33.81	22.77	73.43	9.86	29.60
*The proportion of T_*Wrc*_*(%)	52.78	54.12	29.77	46.31	78.85	58.61	80.94	45.05	72.98	30.92	61.92	44.33	53.39	78.29	65.03	77.23	26.57	64.23	51.86

*This indicator is only the internal component of calculating the T-value of the inequality between groups. The value is positive or negative, which is different from the T value after summation. Referring to Huang Guoping (2012) (26), the value is rounded off, and the actual value is not zero, so the calculated contribution is not zero. (The table below is the same)*.

#### The Regional Dimension Decomposition Results of the Total T-Values

In this paper, the total *T*-value of the equalization of the drug welfare induction level in patients with chronic diseases is calculated as follows: total *T* = 0.0003, which is close to 0. Therefore, the level of the drug welfare response of Chinese patients with chronic diseases is relatively equal overall.

#### The Regional Dimension Decomposition Results of the T-Values of Each Index

**Figure 4:**
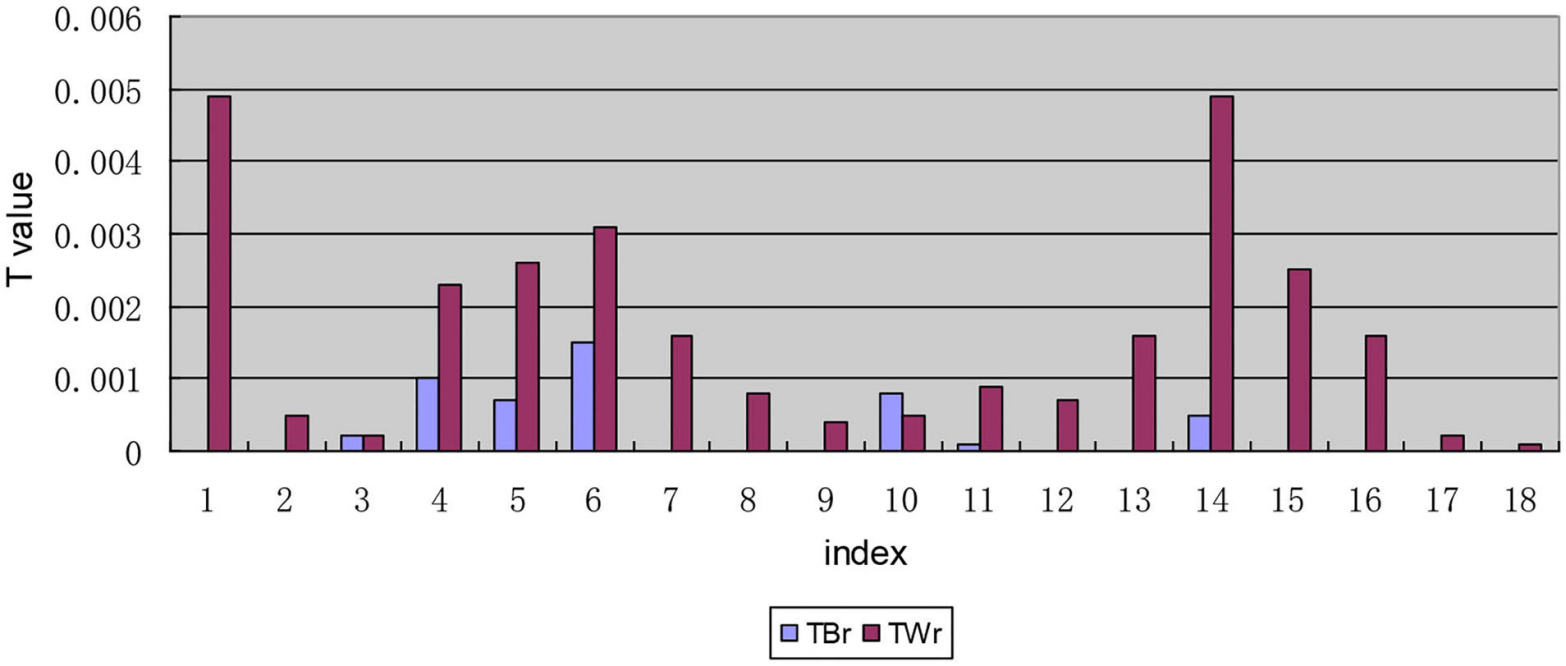
Decomposition of *T-*values of each indicator along the regional dimensional.

After decomposing the *T*-value according to each indicator, the *T*-values of “I1: The number of medical service institutions that can be reached within 15 min,” “I6: The expensive needed drug is not affordable” and “I14: Second reimbursement level of drug expenses” are higher than the other indicators, the values are 0.0050, 0.0046, and 0.0054, respectively, and the *T*-values are all >0.0040 (see Figure 4), which means that the level of equalization of the drug welfare level of these three indicators is relatively low.

#### The Results of the Internal Composition of the Interregional Inequality of the T-Values of Each Indicator

In The internal composition of the interregional inequality, Table 3 lists the weighted logarithm of the mean value of the sensory level of the drug welfare indicators in the patients with chronic diseases in the eastern and western regions during the calculation of the *T*-value of the interregional inequality (the perceived level of welfare is proportional to the total level of welfare induction). Table 3 shows that except for the *T-*values of the indicator “I17: EQ-5D-5L” in the eastern and western regions, the *T*-values of indicators I1, I2, I4-I12, and I14 (see Table 1 for the meanings of the specific indicators, the same below) are all positive in the eastern region, suggesting that the level of drug welfare induction of patients with chronic diseases in the eastern region is at a high level as measured by these indicators. That is, the level of drug welfare induction of patients with chronic diseases in the eastern region is higher than the national average for these indicators. The opposite is found for the indicators above in the western region, and all of their values are negative, indicating that patients with chronic diseases in the western region have less than the national average level of welfare induction in terms of these indicators and therefore a low level of welfare.

#### The Internal Decomposition Results of the Intra-Regional Inequality of the T-Values of Each Indicator

**Figure 5:**
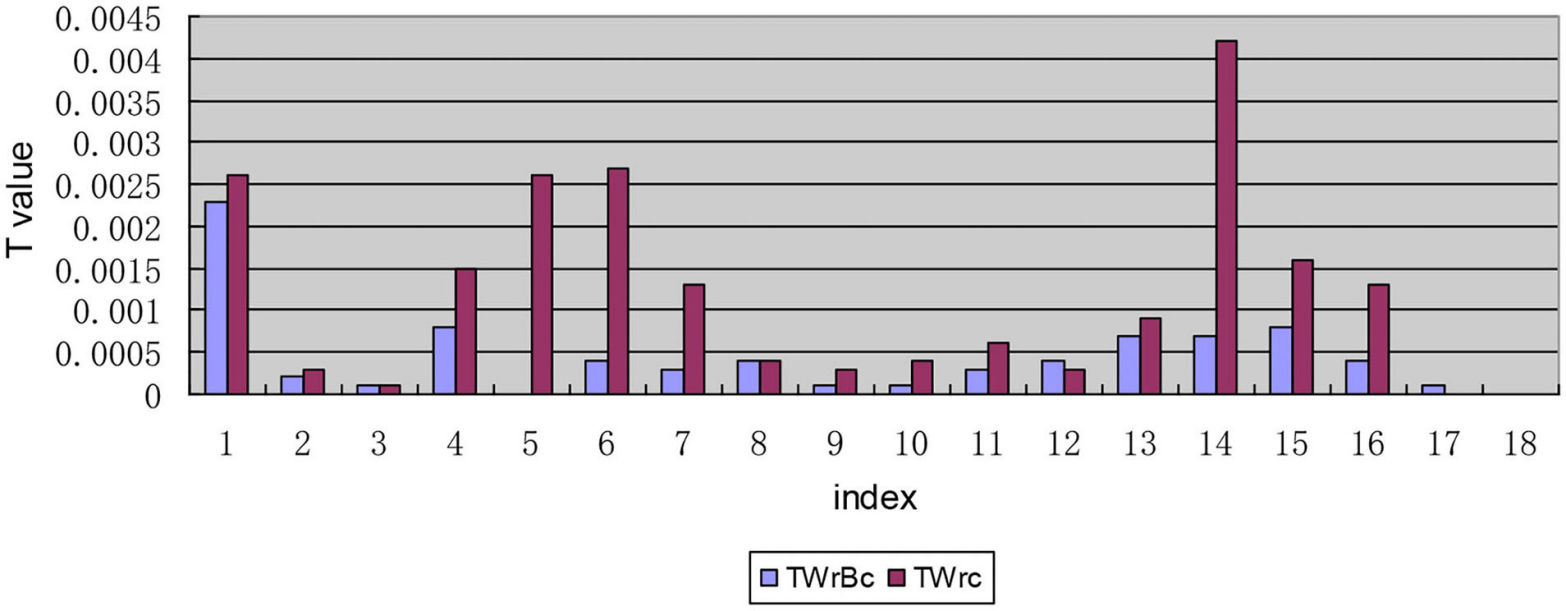
Intra-regional inequality between each indicator decomposition based on the urban and rural dimension.

In the decomposition of intra-regional disparity, the second-order decomposition of the urban and rural dimensions of the indicators “I1: The number of medical service institutions that can be reached within 15 min,” “I6: The expensive drug needed is not affordable,” “I14: Satisfaction with the second reimbursement level for drug expenses,” and “I16: Satisfaction with the rationality of prescriptions by medical institutions” is different from that of the other indicators in the inequality within urban and rural areas within regions, with *T*_*Wrc*_ values >0.0020. Moreover, the inequality within urban and rural areas within regions in these four indicators is larger than the inequality between urban and rural areas within regions (see Figure 5).

### Urban-Rural Dimension: First Bivariate Theil-T Index Hierarchical Decomposition Calculation

By substituting relevant data into formula 2, the bidimensional hierarchical calculation results shown in Table 4 can be obtained. Compared with the decomposition of the bivariate Theil-T index with priority to the regional dimension, the internal composition of the bivariate Theil-T index with priority to the urban and rural dimension has changed.

**Table 4:** Theil index decomposition results of drug welfare induction level of patients with chronic diseases in urban and rural areas.

	**I1**	**I2**	**I3**	**I4**	**I5**	**I6**	**I7**	**I8**	**I9**	**I10**	**I11**	**I12**	**I13**	**I14**	**I15**	**I16**	**I17**	**I18**	**Total**
*T*	0.0050	0.0005	0.0004	0.0033	0.0033	0.0046	0.0016	0.0009	0.0004	0.0013	0.0010	0.0007	0.0016	0.0054	0.0025	0.0016	0.0002	0.0001	0.0003
*T_*Bc*_*	0.0021	0.0000	0.0000	0.0003	0.0000	0.0004	0.0002	0.0001	0.0000	0.0001	0.0000	0.0000	0.0000	0.0000	0.0006	0.0002	0.0000	0.0000	0.0000
Weighted logarithm (urban)	0.0225	−0.0028	0.0015	0.0087	0.0002	0.0089	0.0067	−0.0046	−0.0004	0.0042	−0.0030	−0.0032	0.0004	0.0001	0.0112	−0.0064	−0.0025	−0.0012	0.0017
Weighted logarithm (rural)	−0.0204	0.0029	−0.0015	−0.0084	−0.0002	−0.0086	−0.0065	0.0047	0.0004	−0.0041	0.0030	0.0032	−0.0004	−0.0001	−0.0106	0.0066	0.0025	0.0012	−0.0017
*T_*Wc*_*	0.0028	0.0004	0.0004	0.0030	0.0033	0.0042	0.0014	0.0008	0.0004	0.0012	0.0010	0.0007	0.0016	0.0054	0.0019	0.0014	0.0001	0.0001	0.0003
*T_*WcBr*_*	0.0002	0.0002	0.0003	0.0015	0.0007	0.0016	0.0001	0.0004	0.0001	0.0008	0.0003	0.0004	0.0008	0.0012	0.0003	0.0002	0.0001	0.0000	0.0001
Interarea within the urban	0.0002	0.0001	0.0000	0.0001	0.0004	0.0008	0.0001	0.0003	0.0001	0.0003	0.0003	0.0002	0.0002	0.0012	0.0001	0.0001	0.0000	0.0000	0.0001
Interarea within the rural	0.0000	0.0000	0.0002	0.0014	0.0003	0.0007	0.0000	0.0001	0.0000	0.0005	0.0000	0.0002	0.0005	0.0000	0.0003	0.0001	0.0000	0.0000	0.0000
*T_*Wcr*_*	0.0026	0.0003	0.0001	0.0015	0.0026	0.0027	0.0013	0.0004	0.0003	0.0004	0.0006	0.0003	0.0009	0.0042	0.0016	0.0013	0.0000	0.0000	0.0002
Eastern interior	0.0025	0.0002	0.0000	0.0006	0.0020	0.0011	0.0013	0.0001	0.0002	0.0003	0.0001	0.0002	0.0004	0.0040	0.0010	0.0010	0.0000	0.0000	0.0000
Eastern interior of urban areas	0.0002	0.0001	0.0000	0.0003	0.0010	0.0007	0.0000	0.0000	0.0002	0.0001	0.0000	0.0000	0.0001	0.0021	0.0000	0.0005	0.0000	0.0000	0.0000
Eastern interior of rural areas	0.0023	0.0000	0.0000	0.0003	0.0010	0.0005	0.0012	0.0001	0.0000	0.0002	0.0001	0.0002	0.0003	0.0019	0.0010	0.0005	0.0000	0.0000	0.0000
Western interior	0.0001	0.0001	0.0001	0.0009	0.0006	0.0016	0.0000	0.0002	0.0002	0.0001	0.0005	0.0001	0.0005	0.0002	0.0006	0.0003	0.0000	0.0000	0.0001
Western interior of urban areas	0.0001	0.0001	0.0000	0.0000	0.0005	0.0011	0.0000	0.0002	0.0000	0.0000	0.0004	0.0000	0.0001	0.0001	0.0006	0.0003	0.0000	0.0000	0.0001
Western interior of rural areas	0.0000	0.0000	0.0001	0.0009	0.0001	0.0005	0.0000	0.0000	0.0001	0.0000	0.0001	0.0001	0.0004	0.0000	0.0001	0.0000	0.0000	0.0000	0.0000
*The proportion of T_*Bc*_*(%)	43.14	8.02	2.54	10.14	0.00	7.72	12.33	11.67	0.16	6.11	4.22	6.41	0.04	0.00	22.03	12.01	17.50	8.88	4.26
*The proportion of T_*Wc*_*(%)	56.86	91.98	97.46	89.86	100.00	92.28	87.67	88.33	99.84	93.89	95.78	93.59	99.96	100.00	77.97	87.99	82.50	91.12	95.74
*The proportion of T_*WcBc*_*(%)	4.08	37.86	67.69	43.55	21.15	33.68	6.73	43.28	26.85	62.97	33.87	49.26	46.57	21.71	12.94	10.76	55.93	26.89	43.88
*The proportion of T_*Wcr*_*(%)	52.78	54.12	29.77	46.31	78.85	58.61	80.94	45.05	72.98	30.92	61.92	44.33	53.39	78.29	65.03	77.23	26.57	64.23	51.86

#### Regional Dimension Decomposition Results of Total T-Values

The results of the total *T-*values obtained by the decomposition of the urban and rural dimension are the same as those obtained by the decomposition of the regional dimension.

#### Decomposition Results of T-Values of Each Indicator Along the Urban-Rural Dimension

**Figure 6:**
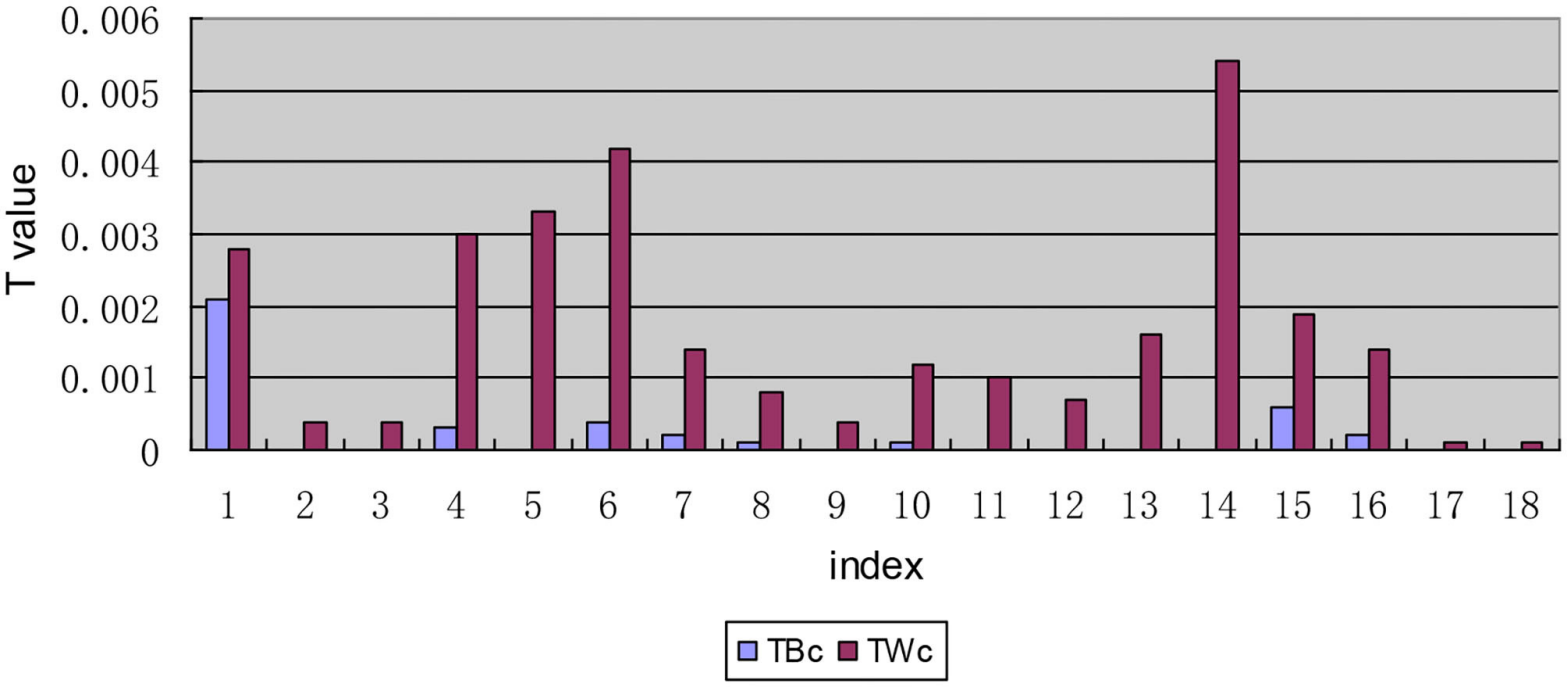
Decomposition of *T-*values of each indicator along the urban and rural dimension.

After the first-order decomposition of each indicator along the urban-rural dimension, the comparison of *T*-values between urban-rural areas and within urban-rural areas shows that the *T-*values of I1, I4, I5, I6, and I14 of the equalization of drug and welfare response levels for Chinese patients with chronic diseases are significantly different from the values for other indicators. On the whole, China needs to increase the accessibility and fairness of drug distribution in terms of improving the level of drug welfare of patients with chronic diseases, especially in terms of the accessibility of medicines. Furthermore, in addition to the two indicators of I17 and I18, the inequality within urban and rural areas in the remaining indicators is larger than that between urban and rural areas. A visual comparison of the inequality between urban and rural areas and the inequality within urban and rural areas is shown in Figure 6.

#### Internal Composition of the Inequality Between Urban and Rural Areas

In the internal composition of the inequality between urban and rural areas, Table 4 lists the weighted log values of the deviation of the induction level from the average value of all indicators in the process of calculating the *T*-value of the inequality between urban and rural areas. In the composition of the *T*_*Bc*_ value, the urban component value of indicators I1, I3-I7, I10, and I13-15 is positive, while the rural component value is negative, indicating that these indicators are low in the rural area. The urban component value of indicators I2, I8, I9, I11, I12, and I16-I18 is negative, while the rural component value is positive. Therefore, these indicators are at a low level in cities.

#### The Internal Composition of the Inequality Within Urban and Rural Areas

**Figure 7:**
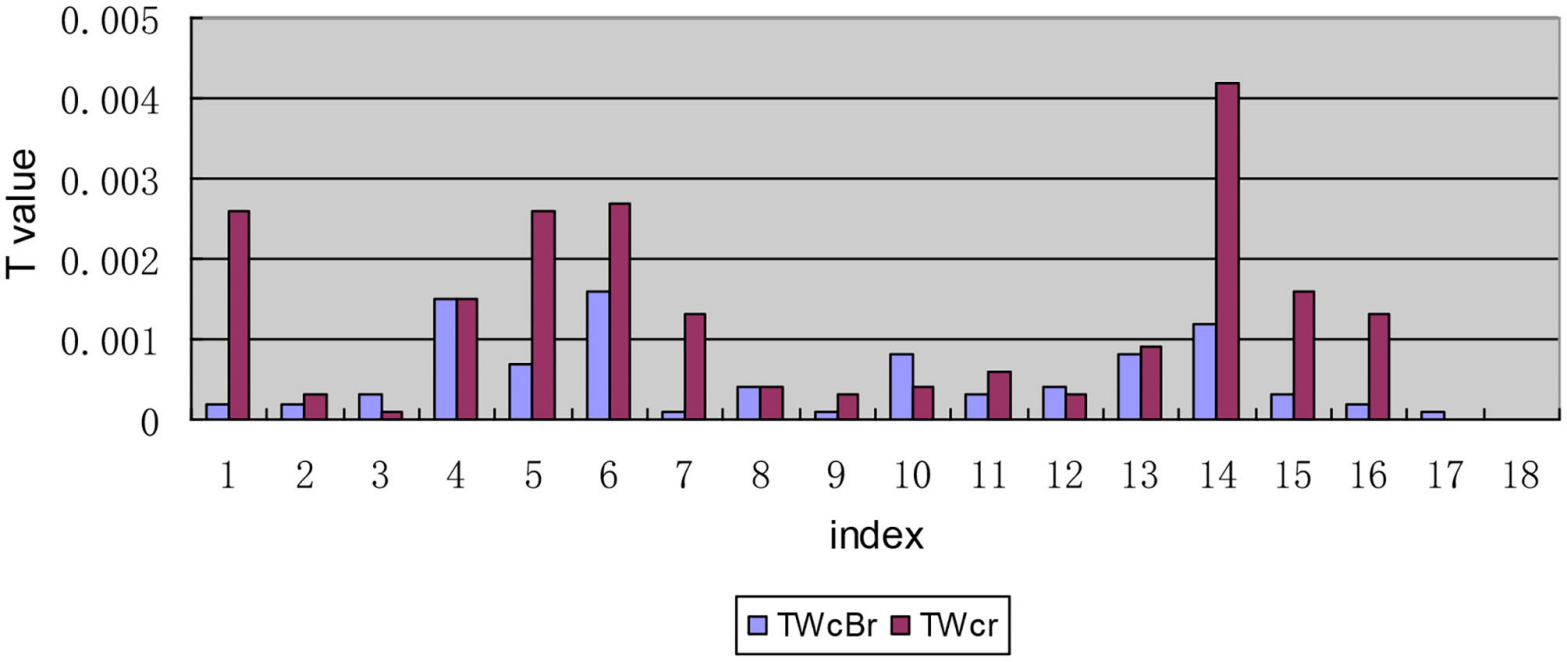
Decomposition diagram of the inequality in each indicator between urban and rural areas by region.

**Figure 8:**
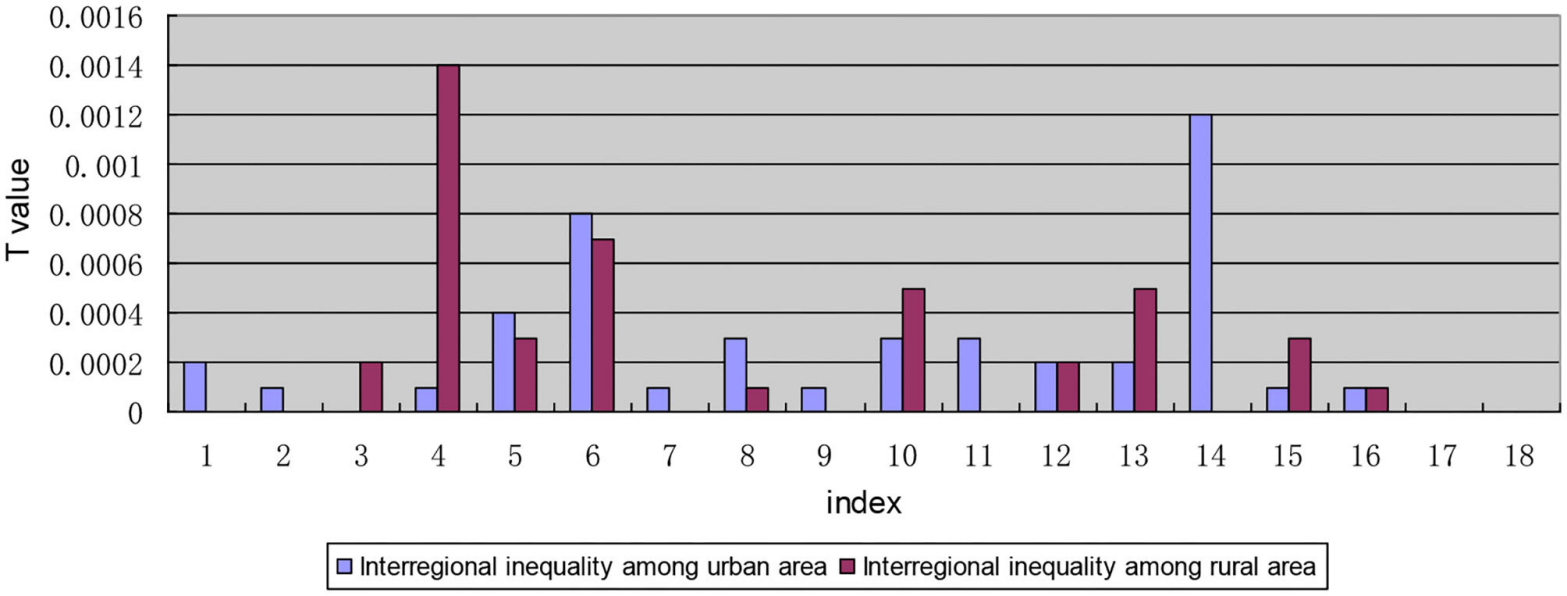
Internal composition of the interregional inequality for each indicator within urban and rural areas.

In the composition of the inequality within urban and rural areas, the intra-regional inequality within urban and rural areas found for indicators I1, I5, I6, and I14 are significantly higher than those the interregional inequality within urban and rural areas (see Figure 7). Further analysis of the interregional inequality within urban and rural areas shows that the inequality between rural areas for the five indicators of I3, I4, I10, I13, and I15 is larger than that between urban areas (see Figure 8).

**Figure 9:**
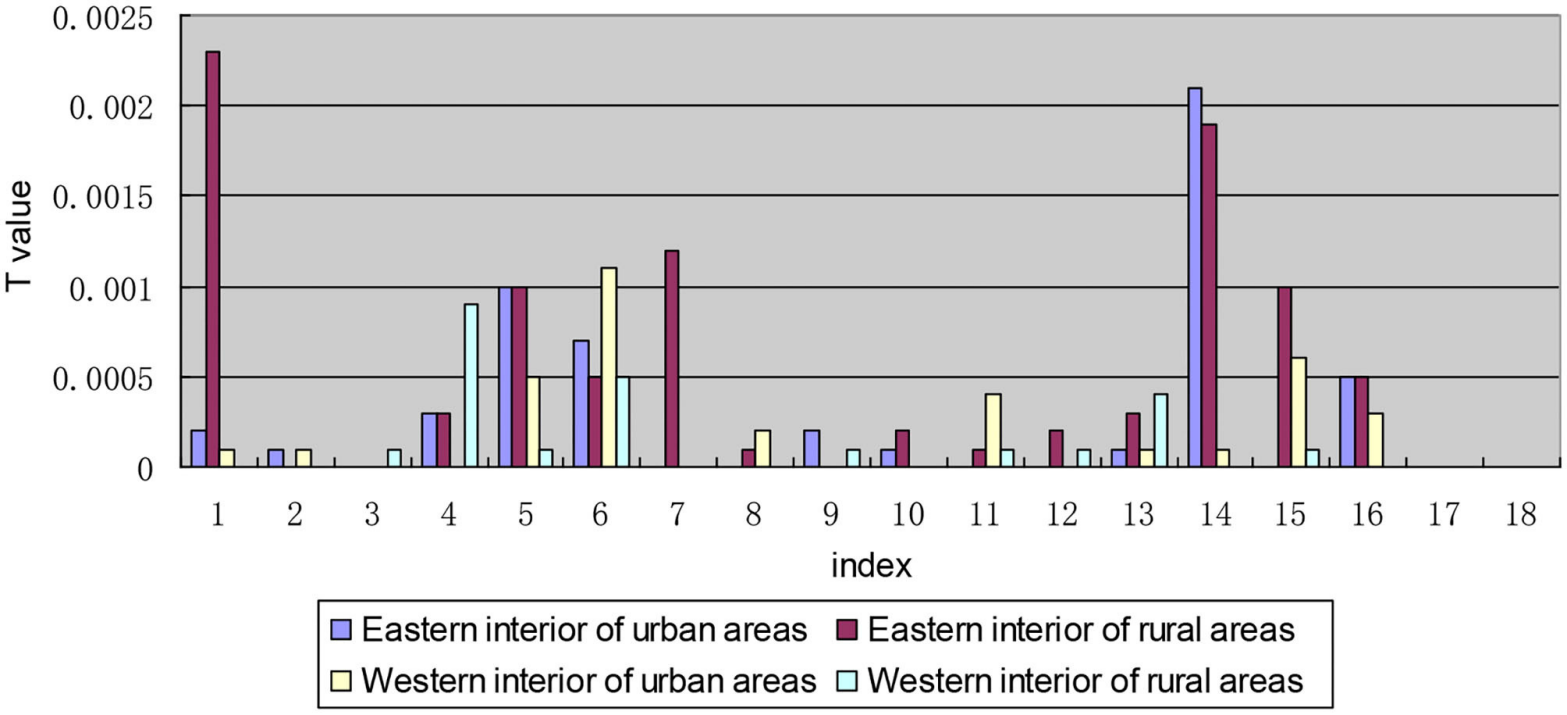
Internal composition of the intra-regional inequality within urban and rural areas.

Meanwhile, It can be seen from the composition of the intra-regional inequality within urban and rural areas (Figure 9) that the indicators “I1: satisfaction with the number of medical and health service institutions within 15 min,” “I7: proportion of drug expenditure in household disposable income,” and “I15: affordable level of out-of-pocket drug expenses” show a large inequality in rural areas in eastern China. For indicators “I4: the inequality between rural areas in western China is large when the required drug is available in online drug stores,” “I5: unavailability of cheap drug needed,” and “I14: satisfaction with the level of secondary reimbursement of drug costs,” the rural-eastern internal inequality is similar to the urban-eastern internal inequality, and the inequality is larger than that for other indicators. Indicator “I6: The expensive drug needed is not affordable” shows a large inequality in the urban areas in the western region. The reasons for the above phenomenon are mainly found in two aspects: on one hand, the disparity of economic development level among provinces in the region is still large; on the other hand, local governments pay different levels of attention to drug welfare and support it in different ways. While the state is committed to solving the inequality of drug welfare between urban and rural areas, it pays insufficient attention to the intra-regional inequality within urban and rural areas.

## Discussion

In this study, we construct an indicator system to measure the drug welfare induction level of patients with chronic diseases in China. We use the bivariate Theil index for hierarchical decomposition to empirically analyze the samples of four provinces under investigation and find out the differential structure of the drug welfare induction level of patients with chronic diseases from the regional dimension and the urban-rural dimension.

In the decomposition results of both regional dimension priority and urban-rural dimension priority, the total T value of the drug welfare induction level is 0.0003, which is close to 0, indicating the overall induction level is relatively equal. This shows that the differences of chronic disease drug management and health services in China are not great as a whole. There is not much difference in the medication situation of patients, which is similar to the conclusions of previous studies: The vast majority of CNCD patients do not receive proper care, and more than 70% of CNCD patients are not well-controlled (40). From 2010 to 2014, the gap of self-rated health between different income groups in China had been narrowed and health equity had been improved (41).

### Bivariate Theil-T Index With Regional Dimension Priority

The hierarchical decomposition of the Theil index with priority in the regional dimension shows that the intra-regional (eastern region interior or western region interior) gap of the drug welfare induction level of patients with chronic diseases in China is larger than the inter-regional (between eastern and western regions) gap. This is because China's current poverty alleviation policies for health in the west have gradually narrowed the gap between regions. For example, the government has increased the proportion of subsidies to the central and western regions and remote and poor areas when arranging subsidy funds for public health and resident medical insurance; it also has implemented special subsidies for poor areas and focused on improving the medical and health service capabilities of the central and western regions. Unlike previous conclusions, previous studies have shown that there are significant regional differences in the distribution of health resources in China (42). The *T*-value of the index I1 is > 0.0040 and 0.0020 after the first decomposition and the second decomposition, respectively, indicating that the level of induction of chronic disease patients represented by this index is relatively low. In other words, the degree of equalization of access to medical and health institutions is not high. It is worth noting that in the primary and secondary decomposition results of the regional dimension priority, the *T-values* of indicators I6 and I14 are larger than that of others, indicating that patients' induction of the affordability of high-priced drugs and the fairness of secondary reimbursement of drug costs is quite unbalanced. Previous studies have shown that in terms of per capita consumption capacity, the price of new anticancer drugs is unaffordable in China (43).

In terms of the internal composition of the inter-regional gap, patients with chronic diseases in the eastern region have a higher level of drug welfare induction, which is consistent with national conditions and related research results (44). The eastern region is economically developed, and the medical insurance fund guarantee is fundamentally stronger than that of the western region. Even if the western region has the support of Health Poverty Alleviation Policy, there is an imbalance in the drug welfare of patients with chronic diseases between the east and west of China, and there is a certain gap.

The price of medicine has always been a major issue related to the national economy and people's livelihood. In recent years, the Chinese government has made tremendous efforts to reduce drug prices and achieved certain results. For example, a series of comprehensive policies have been implemented, including “zero tariffs on imported anti-cancer drugs,” “consistency evaluation and quality evaluation of generic drugs,” “4+7' centralized drug procurement,” and “zero-profit drug policy.” These measures reduce the intermediate links of drug circulation, saving a lot of costs in promotion, distribution and other links, and reduce drug prices to the greatest extent. These drug policies have something in common with other countries: the US federal and state policies on generic drugs have reduced consumer spending; the combined drug procurement program of federal agencies will reduce the average cost sharing of Medicare beneficiaries (45, 46). However, the above-mentioned policies also bring some hidden worries, such as the risk that the burden of patients' medical expenses and the burden of medical insurance fund expenditures will not decrease but increase (47). Through field investigations and empirical analysis, it is found that the current situation of unaffordable high-priced drugs is still grim in both urban and rural areas in China.

### Bivariate Theil-T Index With Urban-Rural Dimension Priority

The hierarchical decomposition of Theil index with priority in the urban-rural dimension shows that the inequality within urban and rural areas in patients' drug welfare induction is much larger than that between urban and rural areas. The contribution rate of the gap within urban and rural areas accounts for 95.74%, which is the main aspect of the inequality of drug welfare for patients with chronic diseases. Early research found that compared with eastern and central regions, the medical and health burdens of farmers in western China (11 provinces and regions) were gradual (48). In particular, the degree of medical equalization in the eastern rural areas is relatively low, which is mainly reflected in the accessibility of medical institutions, the affordability of self-paid drugs, and the proportion of drug expenditures to household disposable income.

The first-order decomposition results show that the gap within urban and rural areas is greater than the gap between urban and rural areas. Although the country is committed to solving the problem of drug inequality between urban and rural areas, it has not paid enough attention to inequality within urban and rural areas. By addressing health inequality and creating a healthy social environment, China will be able to better cope with the heavy burden of chronic diseases (49). The accessibility of medicines represented by indicators I1, I4, I5, and I6 and the fairness of medicines represented by index I14 have a low degree of equalization. It can be seen that the problem of accessibility of medicines is the main aspect of the inequality of drug welfare. Timely and adequate supply of medicines is a difficulty faced by chronically ill patients in rural areas in China. However, under the severe burden of disease, “patients are poor because of diseases, and patients go back into poverty because of diseases.” The composition of the inequality within urban and rural areas shows that the intra-regional inequality in drug welfare for patients with chronic diseases is greater than interregional inequality regardless of whether it is within cities or rural areas. The internal inequality in the west is greater than that in the east, which are mainly embodied in the accessibility of medicines. This is also due to the large gap in the economic development level of the provinces within the region. The low drug welfare in western China is mainly manifested in the poor accessibility of online drugs, which is related to the backward rural economy, lower Internet penetration rate, older chronic disease patients, lower education level, and less popularity of network usage. And it is more difficult to distribute drugs in the western region than that in the eastern region.

### Policy Implications

Based on the above analysis, we seriously recommend that drug policy makers consider our results and formulate relevant intervention measures to improve the level of equalization of drug welfare for patients with chronic diseases.

First of all, in the face of poor affordability of high-priced drugs for chronic disease patients (I6), and whether in urban or rural areas, the intra-regional inequality is significantly larger than the inter-regional inequality, strong medical reform measures should be taken to strengthen the price regulation of commonly used drugs and high-priced drugs. In fact, there are inequalities in drug welfare in all regions of the country, and as far as western provinces are concerned, the driving force of medical insurance policies on the drug welfare of local patients with chronic diseases is also different. The National Healthcare Security Administration, which is directly under the State Council, was established in 2018, avoiding the problem of “decentralized functions” in the past. It enhances the coordination and leadership of medical reform, enabling medical insurance to act as a payer to leverage the transformation of medical service models and forces the reform of the pharmaceutical industry system. A series of policies such as medical insurance fund supervision, mass purchase, medical insurance catalog adjustment, DRG (Diagnosis Related Groups) payment, etc. ensure patients' high-priced drug use, great reduction of the economic burden of patients, improvement of public health, and creation of economic value (50).

Second, in order to ensure the supply of cheap drugs (I5) and the quality and level of medications for patients with chronic diseases, the production and supply guarantee mechanism for cheap drugs should be improved to promote the equalization of cheap drugs in urban and rural areas. Studies have shown that although the industries in developing countries are booming, the poor still cannot afford drugs (51), so the government should ensure the supply of cheap drugs, such as generic drugs (50). On the one hand, the shortage of cheap drugs is due to the low-price drug bidding system, which causes all links of the supply chain to lose production and sales momentum due to lack of profit; On the other hand, the raw materials are stockpiled and scarce, companies that successfully won the bid can only reduce costs, leading to a decline in drug quality. The shortage of cheap medicines at the grassroots level has a major impact on chronically ill patients and doctors (52). Therefore, it is necessary to establish a safety early warning and emergency management mechanism for drug supply.

Third, the government should establish a public health insurance budget system to promote the equalization of secondary drug reimbursements in urban and rural areas (53). Through the above empirical research, it is found that the level of secondary reimbursement of drug expenses for patients in China is still unequal. It is suggested that the existing decentralized basic medical security system be integrated into the province-based single payment system through the reorganization of the financing system, payment structure, and operation mode (54). The government can also promote Urban and Rural Residents' Serious Illness Insurance Livelihood Project, and magnify the superimposed effects and security guarantee effects of various security systems to eliminate the phenomenon of “Poverty due to illness.” In addition, in order to effectively improve the drug welfare of patients with chronic diseases and reduce the burden of outpatient drug costs for patients with hypertension, diabetes, etc., the National Medical Insurance Bureau should establish a complete outpatient medical expenses mutual assistance mechanism and include outpatient medical expenses into the payment scope of the basic medical insurance coordinating fund.

Fourth, the government should strengthen the standardized management of online pharmacies (55) to improve the efficiency of real-time supervision and to enhance patients' trust in Internet drug platforms. Previous studies have shown that purchasing drugs from online pharmacies can improve the accessibility of drugs. However, due to the imperfect supervision system, the illegal sales of prescription drugs have been intensified. Therefore, it is necessary to strictly regulate online pharmacy sales practices (56). At the same time, this study finds that patients in rural areas in western China rarely buy drugs online, which is also related to the current Internet drug sales restrictions in China. Many drugs for the treatment of chronic diseases, such as cardiovascular and cerebrovascular drugs, are basically prescription drugs, while the drugs sold in online pharmacies can only be non-prescription drugs. At the same time, the medical insurance system cannot adapt to medical e-commerce payment, obliterating the convenience and price advantage of online drugs. The “Internet+” model should innovate according to the existing international mature model (57). It is strongly recommended that electronic prescription review methods be promoted in a timely manner and restrictions on online sales of prescription drugs be released on the basis of ensuring drug safety. In addition, the medical e-commerce should be covered by medical insurance system.

### Limitations and Future Research

This study has some limitations. First, the patient's profile and environmental factors may vary geographically. Therefore, the environment of these four provinces may not represent all urban and rural areas in China. Second, the sensitivity level of drug welfare is easily affected by other factors, such as the regional economic system and the personal qualities of patients. Therefore, longitudinal studies with large sample sizes are expected to further explore the determinants that affect the induction level equalization.

## Conclusion

As the population of chronic diseases in China continues to expand, our research results indicate that the government should focus on increasing the level of drug welfare for chronic disease patients in the western rural areas in the future. The policy should be tilted toward the disadvantaged provinces within the region to promote the equalization of the level of drug welfare for patients with chronic diseases in China.

## Strengths and Limitations of This Study

There was little previous research on the equalization of drug welfare induction level of Chinese patients with chronic diseases.

We set up the index system of drug welfare level by using the questionnaire, and studied it from four aspects of Drug Accessibility Effect Induction level, Drug Price Effect Induction leve, Drug Fairness Effect Induction level, and Drug Health Effect Induction level.

We Creatively used the bivariate Theil-T index method to measure the level of drug welfare induction.

Limitations include the subjectivity of the indicator system and the representativeness of the sample.

## Data Availability Statement

The datasets used or analyzed in the current study are available from the corresponding author upon reasonable request.

## Ethics Statement

Ethical approval (case reference number: KY2017039) was obtained from the Ethics Committee of Nanjing Hospital of Chinese Medicine affiliated with Nanjing University of Chinese Medicine in China, which undertook the study. Informed consent was obtained in written form. All subjects gave their informed consent for inclusion before they participated in the study.

## Author Contributions

ST and RZ: designing the manuscript and structure and writing the manuscript. YS: data processing and collected the references. YG: indicator calculation and result analysis. YC: checked the data and edited the language. All authors contributed to the interpretation of the data and approved the final version for submission.

## Conflict of Interest

The authors declare that the research was conducted in the absence of any commercial or financial relationships that could be construed as a potential conflict of interest.

## Abbreviations

BMI: Body mass index
T: Total gap
T_Br_: Gap between regions
T_Bc_: Gap between cities
T_Wr_: Gap within the region
T_Wc_: Gap within urban and rural areas
T_WrBc_: Gap within the region between urban and rural areas
T_Wrc_: Gap within the region within urban and rural areas
T_WcBr_: Gap within urban and rural areas between regions
T_Wcr_: Gap within urban and rural areas within the region.
