# Supplementary material for: Measurement of the Equality of the Drug Welfare Induction Level of Chinese Patients With Chronic Diseases in Gansu, Sichuan, Hebei, and Zhejiang Based on the Bivariate Theil-T Index Method
Source: Front Public Health. 2020 Oct 28;8:581533. doi: 10.3389/fpubh.2020.581533 (PMC7655781; doi:10.3389/fpubh.2020.581533)
Supplement: Supplementary file 1 [file Data_Sheet_1.pdf]

Questionnaire

Survey area: Survey date:

Dear patient:

Hello! We are the investigator of the National Natural Science Foundation of China, based on the research on the optimization of the health and precision poverty alleviation policy based on the improvement of the drug welfare effect of patients with chronic diseases. The main purpose of this survey is to understand the level of drug welfare induction in patients with chronic diseases, to develop relevant health policies for the country, and to improve the level of drug welfare sensing for patients with chronic diseases. All surveys are for statistical analysis only, and we will keep your information confidential in accordance with the relevant provisions of the Statistical Law of the People's Republic of China. I hope that you can answer the following questions truthfully, thank you very much for your support and cooperation!

"The data obtained in the statistical survey that can identify or infer the identity of a single statistical survey object shall not be provided or disclosed to any unit or individual, and may not be used for purposes other than statistics."

《The Statistical Law of the People's Republic of China》 (Chapter 3, Article 25)

Questionnaire list

Table 1. Personal situation questionnaire

Table 2. Drug accessibility questionnaire

Table 3. Drug price effect questionnaire

Table 4. Drug Fair Effect Questionnaire

Table 5. European five-dimensional health scale

Table 1. Personal situation questionnaire

**A. Basic personal information**							
**Investigated member**		01	02	03	04	05	06
1	Hukou nature: (1) Agriculture (2) Non-agricultural						
2	Gender: (1) Male (2) Female						
3	Ethnicity: (1) Han (2) Zhuang (3) Hui (4) Manchu (5) Others						
4	age						
5	Marital status: (1) Unmarried (2) Married (3) Divorced (4) Widowed (5) Others						
6	Education level: (1) Not attended (2) Elementary school (3) Junior high school (4) High school and technical secondary school (5) Junior college (6) Bachelor degree or above						
7	Occupation: (1) Departmental cadres (2) Medical and health (3) Teachers (4) Scientific and technical personnel (5) Finance (6) Company employees (7) Workers (8) Farmers (9) Unemployed (10) Retired (11) Housework (12) other						
8	Average monthly income(yuan): (1) 500 or less (2) 500-1000 (3) 1000-1500 (4) 1500-2000 (5) 2000-2500 (6) 2500-3000 (7) 3000 or more						
9	Which of the following types of medical insurance you have participated in: (1) basic medical insurance for urban workers (2) basic medical insurance for urban residents (3) new rural cooperative medical care (4) cooperative medical insurance for						

	urban and rural residents (5) commercial medical insurance (6) other						
10	Employment status: (1) Employment (including flexible employment) (2) Retirement (3) School students (4) Unemployed (5) Unemployed						
11	What is your current main source of income? (Multiple choices) (1) Wages (2) Your own retirement pension (3) Peer supply (4) Child supply (5) Other relatives (6) Government assistance (7) Social pension Insurance money (8) other						
12	Do you have a health file? (1) Yes (2) No (3) Don't know						
13	Have you had a health checkup in the last 12 months? (excluding inspections due to illness)<br>(1) Yes (2) No						
14	What is the name of the chronic disease you have diagnosed by your doctor (fill in the number)?<br>Disease number:<br>Respiratory system: 01) tuberculosis 02) chronic bronchitis 03) emphysema and asthma 04) sand lung or pneumoconiosis<br>Upper digestive system: 11) chronic pharyngitis, esophagitis 12) stomach, duodenal ulcer 13) gastritis 14) gastric polyps<br>Hepatobiliary organs: 21) HBS-Ag surface antigen carrying 22) viral hepatitis 23) Liver disease and cirrhosis 24) Schistosomiasis 25) Gallstone disease 26) Chronic cholecystitis<br>Lower digestive system: 31) intestinal polyps 32) chronic colorectal inflammation 33) acute and chronic appendicitis<br>Reproductive system diseases: 41) reproductive system inflammation ((a vagina b cervix c pelvic cavity) 42) uterus fibroids 43) cervical cancer 44) ovarian cancer<br>45) Endometriosis 46) Uterus, ovary, fallopian tube surgery						

	Other systems: 51) Hyperthyroidism 52) Hypertension 53) Coronary heart disease 54) Cerebrovascular disease 61) Others (system diseases not mentioned in the above coding)<br>Malignant tumors: 71) breast cancer 72) colorectal cancer 73) liver cancer 74) lung cancer 55) diabetes 75) gastric cancer 76) other tumors						

Table 2. Drug accessibility questionnaire

This scale consists of 6 questions. Each question has a scale divided into 10 scales. Please rate according to your own situation!

**Investigated member**		01	02	03	04	05	06
1	Are you satisfied with the number of health care providers you can reach within 15 minutes?<br>Very dissatisfied with 0--1--2--3--4--5--6--7--8--9--10 very satisfied						
2	Are the drugs needed available at public health institutions?<br>Very inconsistent with 0--1--2--3--4--5--6--7--8--9--10 very consistent						
3	Can you buy the drugs you need at a retail pharmacy?<br>Very inconsistent with 0--1--2--3--4--5--6--7--8--9--10 very consistent						
4	Can you buy the medicine you need at an online pharmacy?<br>Very inconsistent with 0--1--2--3--4--5--6--7--8--9--10 very consistent						
5	The number of times that cheap drugs are not available<br>Very much 0--1--2--3--4--5--6--7--8--9--10 very much						
6	The number of times the high-priced drugs are not available<br>Very much 0--1--2--3--4--5--6--7--8--9--10 very much						

Table 3. Drug price effect questionnaire

This scale consists of 6 questions. Each question has a scale divided into 10 scales. Please rate according to your own situation!

**Investigated member**		01	02	03	04	05	06
1	Do you think that the proportion of drug expenditures to household disposable income is reasonable?<br>Very unreasonable 0--1--2--3--4--5--6--7--8--9--10 very reasonable						
2	Do you think that the cost of drugs spent in public health care institutions is reasonable?<br>Very unreasonable 0--1--2--3--4--5--6--7--8--9--10 very reasonable						
3	Do you think that the cost of drugs spent at a physical pharmacy is reasonable?<br>Very unreasonable 0--1--2--3--4--5--6--7--8--9--10 very reasonable						
4	Do you think the cost of drugs spent at online pharmacies is reasonable?<br>Very unreasonable 0--1--2--3--4--5--6--7--8--9--10 very reasonable						
5	Do you think your outpatient drug costs are reasonable?<br>Very unreasonable 0--1--2--3--4--5--6--7--8--9--10 very reasonable						
6	Do you think your hospitalization costs are reasonable?<br>Very unreasonable 0--1--2--3--4--5--6--7--8--9--10 very reasonable						

Table 4. Drug Fair Effect Questionnaire

This scale consists of 4 questions. Each question has a scale divided into 10 scales. Please rate according to your own situation!

**Investigated member**		01	02	03	04	05	06
1	Are you satisfied with the level of medical insurance reimbursement?<br>Very dissatisfied with 0--1--2--3--4--5--6--7--8--9--10 very satisfied						
2	Are you satisfied with the level of second reimbursement for drug costs?<br>Very dissatisfied with 0--1--2--3--4--5--6--7--8--9--10 very satisfied						
3	Do you think the burden of medicine costs is heavy?<br>Very heavy 0--1--2--3--4--5--6--7--8--9-10 very light						
4	Are you satisfied with the prescription of the medical institution?<br>Very dissatisfied with 0--1--2--3--4--5--6--7--8--9--10 very satisfied						

Note: The second reimbursement includes reimbursement for major illness insurance, medical assistance, sickness assistance, commercial health insurance and charity assistance.

Table 5. European five-dimensional health scale

**Investigated member**		01	02	03	04	05	06
1	Ability to move: 0 = unable to move around 2.5 = serious difficulty in walking around 5 = moderate difficulty in walking around 7.5 = difficulty in walking around 10 = no difficulty walking around						
2	Self-care: 0=I can't take a bath or dress myself 2.5=I have serious difficulties in taking a bath or dressing 5=I have a moderate difficulty in taking a bath or dressing 7.5=It’s a little difficult to take a bath or dress 10=I don’t have difficulty bathing or dressing myself.						
3	Daily activities: 0 = unable to carry out daily activities 2.5 = serious difficulties in daily activities 5=It is moderately difficult to carry out daily activities. 7.5=It is a little difficult to carry out daily activities. 10=No difficulty in daily activities						
4	Pain or discomfort: 0 = very severe pain or discomfort 2.5 = severe pain or discomfort 5 = moderate pain or discomfort 7.5 = a little pain or discomfort 10 = no pain or discomfort						
5	Anxiety or depression: 0 = very severe anxiety or depression 2.5 = severe anxiety or depression 5 = moderate anxiety or depression 7.5 = a little anxiety or depression 10=No anxiety or depression						
